# Nighttime fears in children: clinical characterisation and the user needs and preferences for a digital parent-led intervention

**DOI:** 10.1186/s13034-025-01011-2

**Published:** 2026-01-21

**Authors:** Melissa Aji, Amy Datyner, Julie Gougeon, Neelesh Paravastu, Stuart L. Champ, Chloe Y. S. Lim, Arthur Teng, Jennifer L. Hudson

**Affiliations:** 1 Black Dog Institute, University of New South Wales, Sydney, NSW Australia; 2 Faculty of Medicine and Health, University of New South Wales, Sydney, NSW Australia; 3 Department of Sleep Medicine, Sydney Children’s Hospital, Sydney, NSW Australia

**Keywords:** Fear, Anxiety disorders, Sleep wake disorders, Child, Digital health

## Abstract

**Background:**

Nighttime fears are highly prevalent and are associated with sleep disturbances and later anxiety disorders. Despite their impact, research and treatment options remain limited. Few studies have examined the clinical profile of this population. Families have few intervention options, especially those that are accessible and evidence-based. This mixed-methods study aimed to [1] assess the clinical profile of children experiencing nighttime fears, and [2] explore parents’ needs and preferences to inform the co-design of a digital, parent-led exposure-based intervention.

**Methods:**

A co-design approach (*N* = 44) was used involving an online assessment battery with parents of children aged 7–12 years experiencing nighttime fears (*n* = 34; M child age = 9.6 years; 56% female), two phases of semi-structured interviews with a separate group of parents (*n* = 5; median child age = 10.7 years; 60% female), and a focus group with psychologists (*n* = 5; median age: 31.0; 60% female). The assessment battery included a diagnostic instrument assessing DSM-5 psychiatric disorders, and a measure of sleep disturbances. Interviews explored both parents’ experiences and their feedback on a prototype intervention. Psychologists discussed clinical insights and treatment approaches. Quantitative data were analysed descriptively; qualitative data were analysed using framework analysis.

**Results:**

Separation Anxiety Disorder (24%) and Specific Phobia – Natural/Environment type (23%) were the most common primary diagnoses. Sleep disturbances were prevalent, with 70% scoring in the pathological range for initiating and maintaining sleep. Qualitative findings revealed significant emotional distress for both children and parents, limited access to care and the need for clear guidance. Psychologists emphasised the central role of parents and the need for structured support.

**Conclusions:**

Children with nighttime fears often meet criteria for psychiatric disorders and experience significant sleep issues. A co-designed and parent-led digital intervention may address a critical gap for families.

**Supplementary Information:**

The online version contains supplementary material available at 10.1186/s13034-025-01011-2.

## Introduction

Nighttime fears are extremely common, impacting up to 85% of children aged 7–12 years [1]. These are a hetereogeneous group of fears that typically arise in the evening or at bedtime, often involving darkness, monsters, intruders, nightmares or being left alone [1, 2]. In this study, “nighttime fears” refer to fears commonly associated with the nighttime context and include both clinically significant and subclinical presentations.Although nighttime fears are generally considered a normative developmental experience, a significant subset of children - approximately one-third - have persistent and debilitating symptoms [1, 3–8]. While the origin of nighttime fears are not yet fully understood, they are believed to be influenced by exposure to negative information (e.g. stories or media that create sense of danger) [4, 9]. Research has linked normative nighttime fears to psychopathology including anxiety disorders [10–12] and sleep problems [4, 5, 13, 14].

The clinical significance of nighttime fears and the diagnostic profiles of this population have received limited research attention. A previous study found that nighttime fears were associated with one or more DSM-III-R anxiety disorders in approximately 11% of children aged 8–13 years with nighttime fears, namely separation anxiety disorder, generalized anxiety disorder and/or specific phobias [4]. Employing a conservative diagnostic approach, a diagnosis was only assigned when the fear content directly corresponded to diagnostic criteria in a limited and predefined set of anxiety disorders (Specific Phobia, Obsessive-Compulsive Disorder, Panic Disorder, Separation Anxiety Disorder and Generalised Anxiety Disorder). In a separate study examining fears more broadly in children aged 4–12 years, and again using a similar conservative diagnostic approach, 23% of children’s fears were found to correspond to at least one anxiety disorder [15]. Both studies used a subset of the anxiety disorders section of the Diagnostic Interview Schedule for Children (DISC) and were therefore limited in scope.

To date, no studies have adopted a broader diagnostic approach to examine a more comprehensive range of psychiatric diagnoses in children with nighttime fears, such as additional anxiety disorders, mood and disruptive behaviour disorders. Lewis et al. highlighted this gap in a systematic review spanning 25 years of research, noting the limited evidence on the diagnostic status of children with nighttime fears [16]. A recent systematic review [17] further emphasized the substantial limitations in assessing sleep-related fears in children, particularly the lack of standardized and validated assessment tools in the field. This study aims to address this gap by examining the diagnostic profiles of children who experience nighttime fears, to better understand their clinical significance. Additionally, this study aims to explore the extent of sleep disturbances, given the high comorbidity between anxiety and sleep [18–20].

Although research into nighttime fears is expanding, there remains a notable scarcity of treatment programs – both in the research literature and accessible to the public. A recent systematic review [16] identified twelve studies over the past 25 years, and nine randomised controlled trials (RCTs) [21–29]. Of the nine RCTS, five used exposure as a therapeutic intervention [21, 22, 26, 27, 29]. Exposure is considered a critical component of Cognitive Behavioural Therapy (CBT), which is the recommended first line of treatment for paediatric anxiety disorders [30, 31]. Exposure techniques involve the child facing their fears and typically includes a range of repeated tasks (like speaking in class, reducing reassurance-seeking questions, or sleeping in the child’s own bed). The five exposure-based interventions reviewed all require some level of in-person therapist involvement which limits their accessibility. These interventions are primarily aimed at younger children (aged 4–8 years), with the exception of Cornwall et al., which focuses on children aged 7–10 years [21]. This is particularly interesting given that children aged 7–12 years have been shown to experience the highest frequency and severity of nighttime fears [1, 3–5], yet few interventions have been developed or evaluated for this age group. Furthermore, most of the interventions evaluated in the nine RCTs have not been made publicly available to families or clinicians, except for a bibliotherapy intervention for young children aged 4–8 years [29, 32], further limiting accessibility. Digital mental health interventions represent a promising avenue for scalable and accessible treatment solutions [33], particularly given their potential to reach diverse populations and reduce barriers to care. Despite the potential benefits of digital interventions, maintaining engagement, uptake and adherence in these programs remain a challenge [34, 35]. Notably, existing interventions for nighttime fears have not employed co-design methodology – a collaborative approach that involves actively and meaningfully engaging with end users in the development process. Co-design is key in fostering inclusivity and improving uptake, engagement and user satisfaction [36, 37]. The aims of the current mixed-methods study were to: (1) examine the clinical profile of children with nighttime fears, and (2) explore the user needs and preferences of parents, to inform the development of a co-designed digital parent-led, exposure-based intervention for children aged 7–12 years with nighttime fears.

## Method

### Design

The mixed methods study included: (1) an online parent-report diagnostic interview (2), a series of 1:1 semi-structured interview with parents of children with nighttime fears, and (3) a focus group with psychologists. This study was approved by the University of New South Wales (UNSW) Ethics Committee (Approval Number: iRECS5935). All participants provided written informed consent to participate in this study and consent was reconfirmed verbally at the start of each interview and the focus group.

### Participants

Participants comprised of (1) parents of children with nighttime fears and (2) registered psychologists recruited in Australia. Inclusion criteria for parents were as follows: (i) parent or guardian of child 7–12 years old, (ii) child experiences nighttime fears (reported by parent), and (iii) only one parent per family, referring to one child. The presence of nighttime fears was evaluated using a single parent-report item, “*Does your child experience nighttime fears? For example*,* this may include but is not limited to a fear of the dark and/or fear of nightmares*,* noises*,* shadows*,* intruders*,* burglars and monsters.”* Parents responded with a yes/no answer. Inclusion criteria for psychologists were as follows: (i) Australian Health Practitioner Regulation Agency (AHPRA) registered psychologist, and (ii) experience in working with anxiety and sleep in children and/or young people.

### Recruitment and procedures

Convenience sampling was employed using social media advertising and emails to the authors’ network of psychologists, and an internal database of participants with lived experience who previously expressed interest in being contacted for future research studies. Participants were recruited between June 2024 and April 2025. All participants (interview, online assessment and focus group) received a $50 gift card for each activity completed, with a maximum of three $50 gift cards.

#### Interviews and focus group

Participants who expressed interest in participating in the two phases of semi-structured interviews and the focus group were emailed the participant information sheet. Parents completed an eligibility screening questionnaire, and written and verbal informed consent was obtained at the beginning of the interview from parents. It was reiterated that their participation is voluntary and that they could stop at any time. Psychologists were provided with the Participant Information Sheet outlining the eligibility criteria. Although eligibility was not directly assessed via questionnaire for psychologists, it was assumed based on participants’ self-selection into the study. Written informed consent was obtained from psychologists, and an opportunity was provided to contact the researchers with any questions prior to the focus group. Verbal re-confirmation of consent for their participation and the use of audio and visual recordings was obtained prior to each interview and the focus group.

#### Online assessment battery

Prospective participants were directed to a link where they were provided with the participant information sheet and completed a brief screening questionnaire. If eligible, they were invited to provide digital consent to complete the online instrument. Participants were able to complete the questions in their own time and take breaks where needed. Participants who participated in the interview were also invited to complete the online assessment battery; however, none completed both.

### Data collection

#### Interviews and focus group

The study team, including clinical psychologists, mental health researchers and a user experience researcher, developed the semi-structured interview and focus group guides (See Additional File 1). Two phases of semi-structured interviews were conducted with parents. The first phase of interviews aimed to guide the development of early prototype of the digital intervention for nighttime fears by exploring the nature of their children’s nighttime fears, the impact of the family and desired treatment components. Phase two of interviews involved presenting and eliciting feedback on the prototype. The parents were presented with a prototype of the early modules of the program via an online interactive platform that closely resembled the browser format intended for the final program. The prototype included sample content such as an overview of the program and psychoeducation around nighttime fears for parents, and navigation features (e.g. directing parents to navigate back to the home page and eliciting feedback on their experience). Parents’ live interaction with the prototype (via screen sharing) was observed. The focus group sought to elicit expert advice from psychologists, including their experience and expertise in working with children with nighttime fears and their use of exposure, sleep treatment and parent training. Psychologists were also presented with nighttime fear exposure game concepts, which were collectively reviewed and further developed through brainstorming. In addition, psychologists provided input on adapting evidence-based strategies for delivery in a parent-led, digital format to ensure feasibility and acceptability.

Demographics were obtained for all participants via email. Interviews and the focus group were held via Zoom between July 2024 and November 2024 and were audio- and video-recorded. Parent interviews (Stage 1) were conducted by the lead author (Clinical psychologist and child mental health researcher; MA) and user experience researcher (Stage 2; SC), and both authors jointly facilitated the focus group. The average duration of the interviews and focus group were 54 and 58 min, respectively. Field notes were taken to facilitate analysis. Verbatim transcripts of interviews and focus group were generated.

#### Online assessment battery

##### Demographics

Demographics were collected from the parent relating to both the parent and child, including age, gender, sex, location, education and income.

##### Nighttime fears

A series of parent-report questions was used to assess the child’s nighttime fears including their chronicity, frequency, nature of fears, severity of fears, and origin of fears. Items relating to fear origin and coping were adapted from previous literature [4].

Full item wording is provided in Additional File 3.

##### Anxiety and related disorders interview schedule for DSM-5 parent version – computerised (ADIS-5-P-COMP)

The ADIS-5-P-COMP is a digital adaptation of the original ADIS-5-P, a diagnostic interview delivered in a digital format with minimal clinician input. The original ADIS-5-C/P [38] is a semi-structured clinical interview conducted by a trained diagnostician to provide diagnoses of the full range of psychiatric disorders in the DSM-5 for children and adolescents.

The ADIS-5-P-COMP contains separate diagnostic modules for the DSM-5 Disorders, including anxiety disorders (Separation Anxiety Disorder, Social Anxiety Disorder, Generalised Anxiety Disorder, Panic Disorder, Specific Phobia, Selective Mutism), Post-Traumatic stress Disorder (PTSD), Obsessive-Compulsive Disorder (OCD), Depressive disorders (Major Depressive Disorder, Persistent Depressive Disorder, Disruptive Mood Dysregulation Disorder), Conduct Disorder, Oppositional Defiant Disorder and Attention Deficit Hyperactivity Disorder (ADHD). Parents are presented with dynamically tailored questions questions using Qualtrics [39]. That is, the survey used display logic to present follow-up questions based on participants’ previous responses. Parents respond using dichotomous yes/no options, qualitative responses as well as ratings of symptom severity, avoidance, interference, and impact, ranging from 0 to 8. Diagnostic algorithms have been built into the platform to allow personalised questions, tailored to the child’s presentation. Parents are also asked about any historical mental health diagnoses, with a selection of diagnoses provided. We included a question to ask about any historical mental health diagnoses, with a selection of diagnoses provided: ADHD, Autism Spectrum Disorder, Anxiety or related disorder, Depression or other Mood Disorder, Behavioural Disorder (such as Conduct Disorder, Oppositional Defiant Disorder), Eating Disorder or Other (free text box to specify).

A trained clinician reviews and scores the responses to develop a diagnostic profile including the primary and comorbid diagnoses, and a clinical severity rating (CSR), based on the severity and impairment ratings provided by the parent and the qualitative information provided. CSR scores range from 0 to 8, with a CSR of 4 and above indicating the presence of a disorder. The ADIS-5-P-Comp has not been used in prior studies. Previous versions of the ADIS-IV-C/P have demonstrated good inter-rater reliability, test-retest reliability, and construct validity [40]. This tool was administered in this study for research purposes only and diagnostic outcomes were not communicated to participants.

##### Sleep disturbances scale for children (SDSC)

The SDSC [41] is a questionnaire developed to evaluate the occurrence of sleep disturbances in children. The SDSC has 26 items loading on 6 subscales: disorders of initiating and maintaining sleep (DIMS), sleep breathing disorders (SBD), disorders of arousal (DA), sleep–wake transition disorders (SWTD), disorders of excessive somnolence (DOES), and sleep hyperhidrosis (SHY). Parents rate their children’s sleep behaviour and disturbances over the last 6 months, using a 5-point scale (“1 = never, 2 = occasionally, 3 = sometimes, 4 = often, 5 = always”). Total scores range from 26 to 130. T-scores (M = 50, SD = 10) are used for each scale and total SDSC score. A T-Score of 70 is considered in the pathological range; while a T-score between 55 and 70 for school age is considered subclinical. The SDSC has demonstrated high internal consistency and test-rest reliability [41].

### Data analysis

Demographics, clinical and quantitative data were analysed using descriptive analysis. Framework analysis was applied to the qualitative data [42] using 5 stages: (1) familiarisation – immersion and reading of transcripts; (2) developing a thematic framework – identifying key issues using a constant comparative approach; (3) indexing – applying the thematic framework; (4) charting – rearranging data according to the thematic framework to compare data within one interview or theme; and (5) mapping and interpretation – defining concepts and associations between themes.

The interviews and focus group were coded inductively by the lead author (MA). A second researcher independently double-coded the entire first phase of parent interviews (CL) and focus group (NP), as well as 40% of the second phase of interviews (NP). Data was coded using NVivo 12 [43]. Both coders independently developed a thematic codebook, which were then synthesised by the lead author into a single comprehensive codebook. Methodological rigour was ensured by adhering to the COREQ checklist for reporting qualitative research [44] (See Additional File 2). A sample size of five parents and five psychologists was determined a priori to be sufficient for the interviews and focus group, respectively, to adequately capture themes for the purposes of designing an intervention. Data saturation was reached following the first three interviews with parents (stage 1) and the first two interviews (stage 2). While data saturation could not be assessed due to the single focus group, the session provided in-depth insights aligned with themes from other data sources.

All ADIS-5-P-COMP responses were reviewed and scored by Clinical Psychologist (MA). Data was reported separately for clinical and subclinical diagnoses. Parent-reported historical diagnoses were not included in the diagnostic results; only current diagnoses identified through the ADIS-5-P- COMP were considered. Interrater reliability was assessed on a randomly selected 26% of the diagnostic interviews. The overall Cohen’s Kappa of 0.80 indicates substantial agreement between the lead author (MA) and senior author (JH) on the presence/absence of all clinical threshold diagnoses.

Three lived experience advisors - a Clinical Psychologist with experience supporting children with nighttime fears and two parents of children with nighttime fears – were involved throughout the broader co-design process. These advisors were not formal study participants and were not included under ethics approval. However, they contributed to the co-design process, reviewed qualitative findings, and provided feedback on the prototype and manuscript. Two of these advisors (Clinical Psychologist and one parent) are co-authors on this paper and provided more in-depth input contributions, including reviewing the results and the manuscript, which have been incorporated into the final version of the manuscript.

## Results

### Online assessment battery

#### Participant characteristics

Thirty-four parents (94% female; M: 40.3) participated, with children averaging 9.6 years, 56% of whom were female. See Table 1 for further demographics.

**Table 1:** Parent and child demographics

Participant Characteristics (*N* = 34)	Value
**Parent**	
Age, mean (SD)	40.3 (4.9)
Aboriginal or Torres Strait Islander, n (%)	4 (12)
Gender, female, n (%)	32 (94)
**Education, n (%)**	
High School or TAFE/Apprenticeship	3 (9)
Certificate/Diploma	9 (26)
Undergraduate	9 (26)
Postgraduate	13 (38)
**Geographic Area, n (%)**	
Metropolitan	23 (68)
Regional/rural	11 (32)
**Annual Household Income, n (%)**	
$0 -$85,999	5 (15)
$86,000-$134,999	9 (26)
$135,000-$237,999	15 (44)
$238,000+	2 (6)
Prefer not to answer	3 (9)
**Child**	
Age, mean (SD)	9.6 (1.5)
Sex (recorded at birth), female, n (%)	19 (56)
Aboriginal or Torres Strait Islander, n (%)	5 (15)
**Schooling, n (%)**	
Public	25 (74)
Catholic	6 (18)
Independent	3 (9)
School days missed due to mental health, n (%)	
None	18 (53)
1–10	12 (35)
11–20	1 (3)
21+	3 (9)
**Parent-reported academic achievement in English and Maths (compared to peers), n (%)**	
Well above average	11 (32)
Above average	6 (18)
Mostly average	10 (29)
Below average	6 (18)
Very varied across subjects	1 (3)

#### Characteristics of children’s nighttime fears

Most children had experienced nighttime fears for a prolonged period. Specifically, 29% had fears lasting 1–3 years, 32% for 4–6 years, and another 29% for 7 years or more. Table 2 presents the characteristics of children’s nighttime fears, including duration, frequency, types of fears and associated factors.

**Table 2:** Characteristics of children’s nighttime fears

Characteristic	*n* (%)
Duration of nighttime fears (years)	
< 1	3 (3)
1–3	10 (29)
4–6	11 (32)
7 +	10 (29)
Frequency of fear	
Often	23 (68)
Sometimes	11 (32)
Known precipitating event	
No	27 (79)
Yes	7 (21)
Exposure to fear-inducing television content	
No	16 (47)
Yes	10 (29)
Maybe	8 (24)
Fears and Worries at Night *	
They worry about the dark	22 (65)
They worry about seeing monsters, ghosts, or other imaginary creatures	17 (50)
They fear real-world dangers like robbers, kidnappers, or murderers	16 (47)
They fear something bad will happen if they are apart from the parent	13 (38)
They think about things that happened during the day or worry about what might happen tomorrow	4 (12)

* Multiple responses allowed

#### ADIS-5-P-COMP

##### Primary diagnoses

As shown in Table 3, the most common primary disorders amongst children with nighttime fears were Separation Anxiety Disorder (24%) and Specific Phobia – Natural/Environmental type (23%), with all identifying darkness as at least one of the feared stimuli. On average, children met criteria for three diagnoses (including anxiety diagnoses and other comorbidities). Among children who met criteria for any psychiatric diagnosis, all were diagnosed with an average of two anxiety diagnoses each. 35% of children had at least one comorbid condition other than anxiety. The majority of children (76%) met criteria for at least one disorder. See Table 4 for frequency and CSR ratings of primary diagnoses.

**Table 3:** Frequency and clinical severity rating (CSR) of primary diagnoses

Primary Diagnoses (*n* = 34)	Frequency, *n* (%)	CSR for Primary Diagnosis, mean (SD)
Separation Anxiety Disorder	8 (24)	4.9 (0.8)
Specific Phobia – Natural/Environmental	6 (18)	4.8 (1.0)
Attention Deficit Hyperactivity Disorder	4 (12)	5.0 (0)
Social Anxiety Disorder	2 (6)	5.5 (2.1)
Specific Phobia – Injury	2 (6)	5.0 (0)
Generalised Anxiety Disorder	1 (3)	6.0
Oppositional Defiant Disorder	1 (3)	5.0
Disruptive Mood Dysregulation Disorder	1 (3)	5.0
Obsessive Compulsive Disorder	1 (3)	5.0
Specific Phobia – Animal	1 (3)	7.0
No diagnosis	7 (21)	NA

*CSR*: Clinical Severity Rating. Percentages are calculated based on the total sample (*N* = 34)

##### All diagnoses

Table 4 presents all diagnoses across participants, including both primary and comorbid conditions, along with their corresponding CSRs.

**Table 4:** Frequency and clinical severity rating (CSR) of all diagnoses

	Frequency, *n* (%)	CSR, mean (SD)
Anxiety Disorders		
Specific Phobia – Natural/Environmental *	21 (62)	4.8 (0.9)
Generalised Anxiety Disorder	14 (41)	4.8 (0.7)
Separation Anxiety Disorder	16 (44)	4.8 (1.0)
Social Anxiety Disorder	12 (35)	4.9 (1.0)
Specific Phobia - Injury	8 (24)	4.0 (1.7)
Specific Phobia – Animal	2 (6)	5.5 (2.1)
Selective Mutism	1 (3)	4 0.0
Other Disorders		
Attention Deficit Hyperactivity Disorder	11 (32)	4.5 (0.7)
Oppositional Defiant Disorder	3 (9)	5.3 (0.6)
Obsessive Compulsive Disorder	2 (6)	4.5 (0.7)
Major Depressive Disorder	2 (6)	4.5 (0.7)
Disruptive Mood Dysregulation Disorder	2 (6)	4.5 (0.7)

Percentages are calculated based on the total sample (*N* = 34). * All participants included fear of the dark among their phobia stimuli

##### Subclinical diagnoses

Subclinical diagnoses were identified across several anxiety disorders. The most common was Specific Phobia – Dark (*n* = 5, 15%), followed by Social Anxiety Disorder (*n* = 4, 12%), Specific Phobia – Injury (*n* = 2, 6%), Generalized Anxiety Disorder (*n* = 2, 6%), and one case each (3%) of Separation Anxiety Disorder, Specific Phobia – Animal, Specific Phobia – Situational, Specific Phobia – Other, and PTSD.

##### Historical diagnoses

Historical parent-reported mental health diagnoses for children included Autism Spectrum Disorder (*n* = 5, 15%), and a single case (*n* = 1, 3%) of an Eating Disorder.

#### Sleep disturbances of children’s nighttime fears

**Fig. 1:**
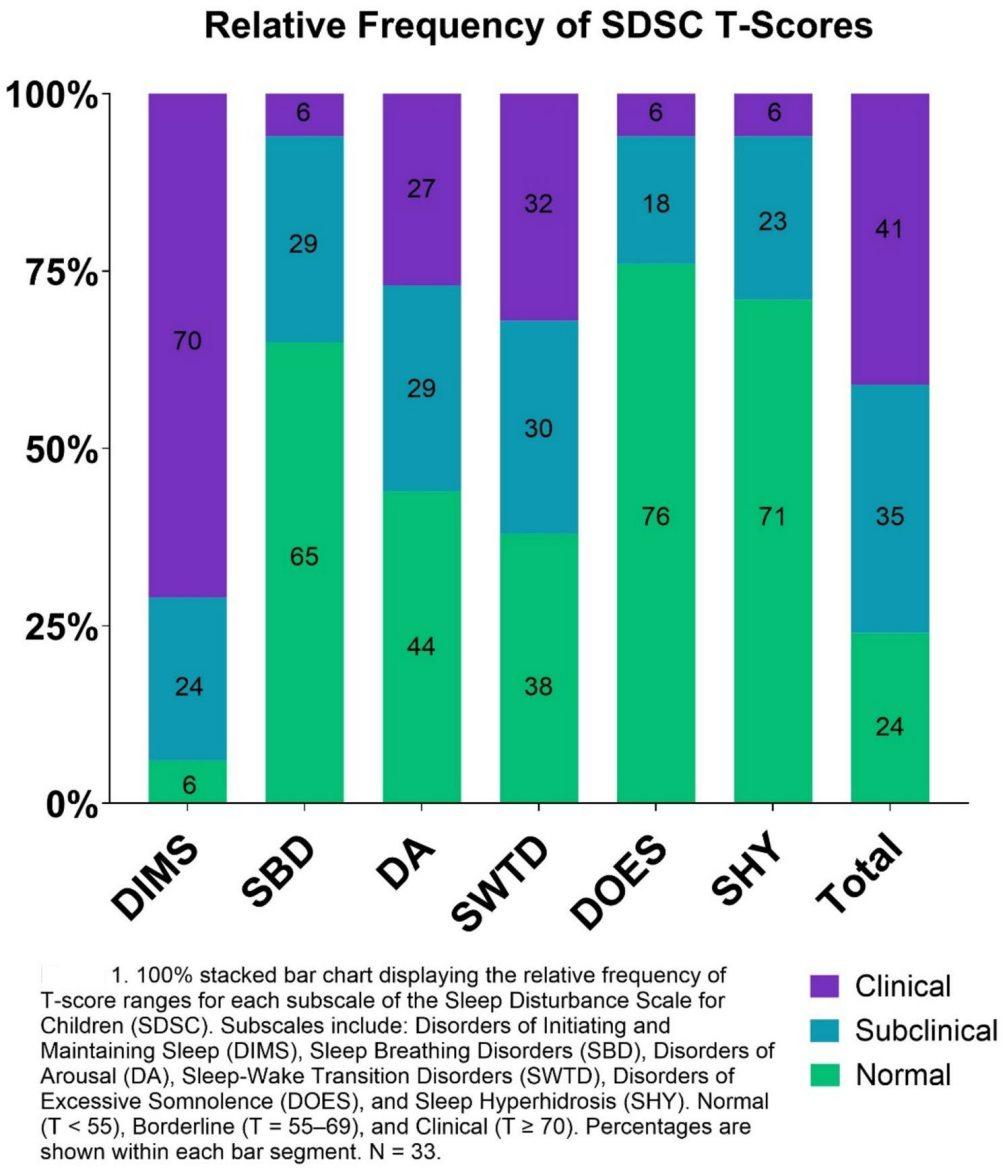
Relative frequency of SDSC T-scores

According to the SDSC total scores, 76% of children fell into the pathological or borderline range indicating the presence of sleep disturbances. 95% of the sample met the threshold for Disorders of Initiating and Maintaining Sleep (DIMS) subscale, with 71% falling into the pathological range and 24% within the borderline range. Over half of children obtained a pathological or borderline score for Sleep-Wake Transition Disorders subscale (SWTD; 62%) and Disorders of Arousal (DA; 56%). Table 5 displays the SDSC T-score subscale scores. Figure 1 presents the relative frequency of SDSC T-scores.

**Table 5:** Sleep disturbance scale for children (SDSC) T-Scores

SDSC T-Score (*N* = 33)	Mean	SD
Disorders of initiating and maintaining sleep (DIMS)	77	15.6
Sleep breathing disorders (SBD)	53	11.9
Disorders of arousal (DA)	60	16.6
Sleep-wake transition disorders (SWTD)	63	14.6
Disorders of excessive somnolence (DOES)	51	9.9
Sleep hyperhidrosis (SHY)	51	9.2
Total	67	13.0

SDSC data were missing for one participant (*n* = 33)

#### Qualitative results

##### Characteristics of parents

**Fig. 2:**
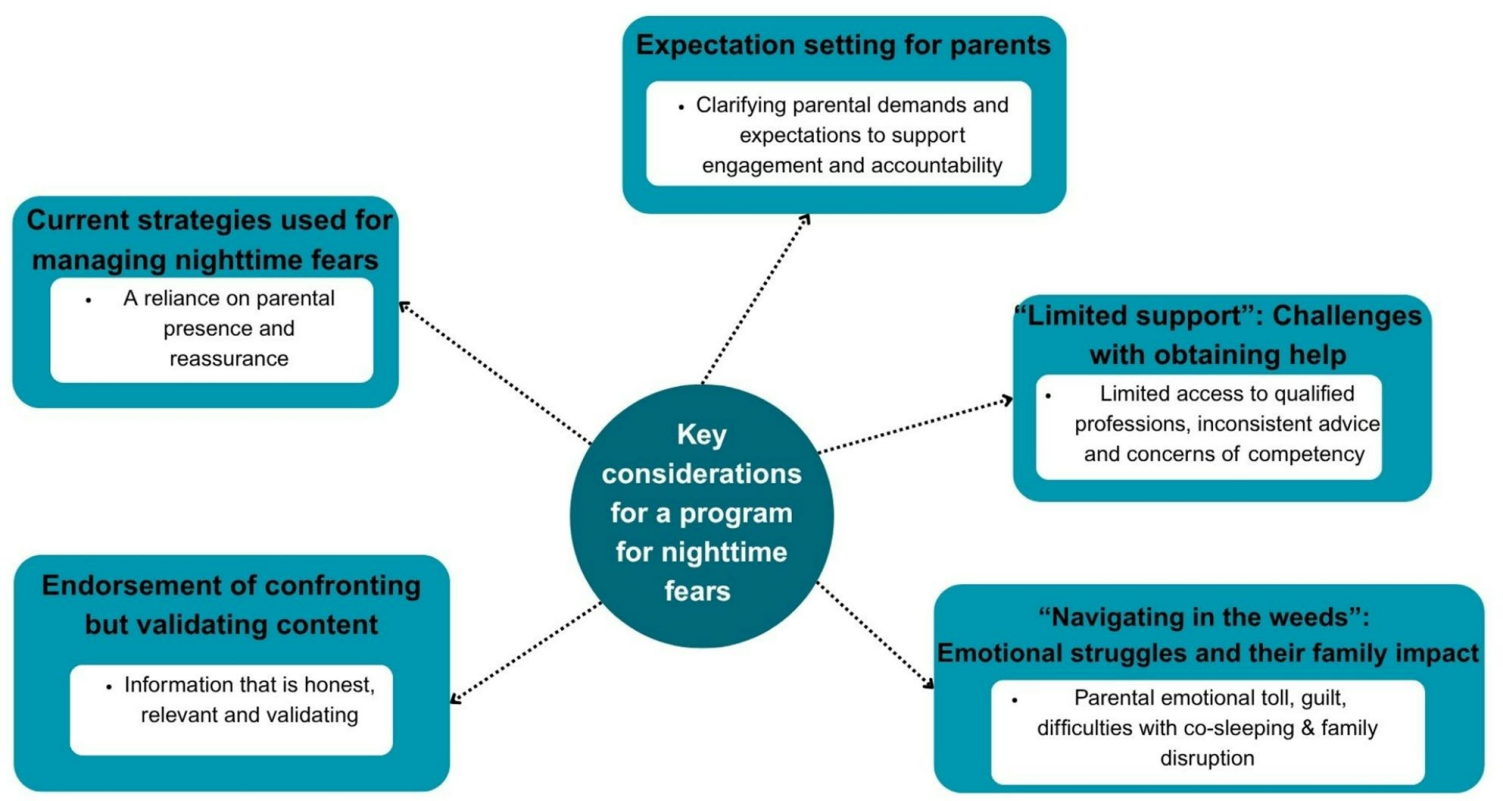
Thematic schema depicting key considerations for a program for nighttime fears

Five parents (age range: 35–51 years; median age: 43.7; 100% female) participated in two phases of interviews. Three parents had a university qualification (60%) and two had completed Year 12 or a certificate/diploma (40%). Most families lived in Metropolitan Australia (60%) and all participants had an annual household income of over $135,000 annually. The median age of children was 10.7 years (range: 10–12) and 60% were female. The majority of children had multiple fears, most commonly a fear of the dark (60%) and imaginary-based creatures e.g. monsters (40%). See Table 6 additional parent demographics details. The overarching themes of the qualitative analysis are represented in Fig. 2.

**Table 6:** Parent-reported demographics (Qualitative Interviews)

Participant characteristics (*N* = 5)	Value
Parent	
Age, median (range)	43.7 (36–51)
Aboriginal or Torres Strait Islander, n (%)	0 (0)
Gender, female, n (%)	5 (100)
Education, n (%)	
High School or Certificate/Diploma	2 (40)
Undergraduate	1 (20)
Postgraduate	2 (40)
Geographic Area, n (%)	
Metropolitan	3 (60)
Regional/rural	2 (40)
Annual Household Income, n (%)	
$135,000-$237,999	5 (100)
Child	
Age, median (range)	10.7 (10–12)
Sex (recorded at birth), female, n (%)	3 (60)
Aboriginal or Torres Strait Islander, n (%)	0 (0)
Nighttime Fear Characteristics	
Duration of nighttime fears (years), m (SD)	3.5 (2.4)
Frequency of Fear	
Often	3 (60)
Sometimes	2 (40)
Nature of Fears	
The dark	3 (60)
Intruders	2 (40)
Imaginary-based creatures	3 (60)
Insects	1 (20)
Death	2 (40)
Fear severity (1–3), m (SD) *	2.6 (0.5)
Known Precipitating Event	
Yes	2 (40)
Exposure to Fear-inducing Media Content	
No	2 (40)
Yes	2 (40)
Maybe	1 (20)

*Where 1 is low severity and 3 is high severity of fears

#### Phase 1: parent interviews

##### Theme 1: current strategies used for managing nighttime fears

Parents reported using a range of strategies to help manage their child’s nighttime fears. All parents reported using reassurance and their presence to help alleviate fears, especially when their child is highly distressed. This included verbal reassurance, lying in bed until their child falls asleep or co-sleeping through the night. Other strategies reported include arousal management strategies, gradual exposure, bedtime routines and reasoning with their fears. Arousal managements strategies included relaxation, audio meditation, music and sleep stories. There were mixed experiences of these strategies reported by parents, with some finding them unhelpful because their child showed little engagement or they were not impactful enough to reduce high level arousal.

##### Theme 2: “Navigating in the weeds”: emotional struggles and their family impact

Parents reported that their child experienced emotional distress and behavioural challenges at night. Common issues included emotional outbursts, such as anger, crying and sadness, as well as behavioural challenges such as parents having difficulty resisting bedtime stalling, and the child clinging to family members.

In response, parents consistently expressed a range of psychological responses including exhaustion, guilt, frustration, agitation, sleep deprivation and anger.

These reflections illustrate the emotional toll of caregiving:

*“It is a huge cost… it’s just navigating in the weeds of feeling angry and resentful and tired” – Parent1*.

“*You are so tired yourself… at your last wits… but then I’d wake up and do all the dishes”- Parent2*.

Many participants spoke of guilt related to their difficulties in supporting their children.

*“When he gets that low*,* I feel like I’ve let him down enormously” [parent crying] – Parent1*.

*“It causes guilt for us. Because we’re like*,* why can’t we give her what she needs?” – Parent4*.

Parents reported varying levels of willingness and capacity to provide repeated and prolonged reassurance or co-sleeping. Parents’ explained:

“*On the nights where he just completely loses it*,* there’s nothing else to do except get into bed with him and hug him. But it also takes a lot. If I’ve had a bad day*,* it’s not the best timing” - Parent1*.

*“just the tears… it’s getting old and tiring. It’s dragged on. Going through the same process and having a struggle every night is not appealing” – Parent5*.

Most parents reported high family conflict at night, often negatively impacting other children in the household and relationships with their spouse. Some parents described worry about the possible persistence of their child’s nighttime fears into later years. Other impacts included occupational with one parent considering leaving her job involving nightshifts to support her child. Another parent spoke of the financial implications, having spent thousands of dollars on babysitters.

##### Theme 3: “limited support”: challenges with obtaining help

Parents described difficulties with access to and the quality of professional help available for nighttime fears. Parents experienced long waiting lists for sleep specialists and mental health practitioners, particularly for those in more remote areas of Australia. In response to being asked what the biggest barrier is to getting support for their child’s nighttime fears, a participant described:

*“It’s the lack of professionals available who are willing to address it… There’s limited people.” – Parent4*.

Parents noted receiving conflicting and unhelpful professional advice, explaining:

*“I’d love to be pointed exactly in the direction of what I should be doing with [my daughter] every night… I’m literally being told completely opposite things by absolute specialists in their field.” – Parent4*.

*“I’ve taken her to a paediatrician. He wasn’t very good*,* though. And he kind of suggested a few ideas*,* and I think he gave me a couple of apps as well. But when I tried to do one of them*,* can’t remember why*,* it wasn’t great. It just didn’t work forever.” – Parent2*.

Only one parent had seen a psychologist and reported this to be unsatisfactory as they were referred back to their doctor:

*“Even with the psychologist*,* even when there was an assessment of the nighttime fears*,* it was more that this needs to be addressed with the physician.” – Parent1*.

#### Phase 2: parent prototype review interviews

##### Theme 1: expectation setting for parents

Many parents expressed a desire for a parent-led program that clearly set expectations from the outset, including providing context of the undertaking, an estimated weekly time commitment and an outline of the possible risks and challenges. Parents noted that by establishing these expectations early, the program can foster a sense of accountability and parental commitment.

*“ [Parents] understand that this is work. This is not a band-aid. It just reinforces the idea that you’re making a commitment to yourself and to your child to see this through” – Parent1*.

For some parents, the desire for setting expectations may have been shaped more by practical constraints or cognitive overload in balancing the program with everyday responsibilities:

“*I might not understand that I can’t multitask my way through this*,* that I can’t be doing this while I’m on a team’s call… I can imagine a number of parents flicking through it to get it done in order to move on… And I think if you want to reinforce the messaging that this is very important” – Parent1*.

For some parents, this need for expectation-setting appeared to come from a place of anxiety and a desire to avoid potential harm:

“*The key that’s really missing from here is what can happen if you don’t do this properly?… the risks if you did this and didn’t read it all*,* and went to night and put your kids to bed without support. They could develop a trauma response” – Parent4*.

##### Theme 2: endorsement of confronting but validating content

Parents were presented with psychoeducational content about nighttime fears, including the nature of fears (e.g. burglars, fear of harm to oneself and loved ones, personal health). Several parents appreciated the inclusion of information that, while confronting, was relevant and accurately reflected and validated their child’s experiences.

*“You’ve got to say it as it is… for my instance*,* my son is incredibly anxious about his own mortality at night*,* and if I didn’t read about that*,* as in personal health or parents dying*,* I would think there was something extreme about his concern… So I think*,* it’s important that we*,* as gently as possible*,* call it exactly what it is” – Parent1*.

*“[The content hasn’t] shied away from actually explaining what [the fears] are like. The burglar – because this is the thing – people avoid talking about it. But this is what the kids are scared of*,* exactly what’s written here. Yeah*,* I’m happy with that… Perfect modelling” – Parent4*.

### Psychologist focus group

#### Characteristics of psychologists

Five AHPRA-registered psychologists (age range: 26–42 years; median age: 31.0; 60% female) participated in an online 1.5 h focus group. The majority of psychologists (80%) had a Masters degree and practiced in a private clinic in a metropolitan area. The majority of participating psychologists (60%) had between 0 and 5 years of clinical practice experience. See Table 7 for additional psychologist demographics details.

**Table 7:** Psychologists’ demographics (Focus group)

Participant Characteristics (Parents; *N* = 5)	Value
Age, median (range)	31.0 (26–42)
Gender, female, n (%)	3 (60)
Education, n (%)	
Masters degree	4 (80)
Doctorate	1 (20)
Geographic Area, n (%)	
Metropolitan	4 (80)
Regional/rural	1 (20)
Type of work *	
Private	4 (80)
Community/Non-Governmental Organisation	3 (60)
Academia	2 (40)
Hospital	2 (40)
School	1 (20)
Years of practice	
0–5	3 (60)
6–10	1 (20)
21–30	1 (20)

*Multiple responses allowed

##### Theme 1: current use of exposure

When asked about current approaches to treating nighttime fears, psychologists described using a range of strategies grounded in Cognitive Behavioural Therapy (CBT), particularly emphasising the use of exposure. Many referenced the use of the “Cool Kids” program including a step-ladder approach, gradually removing safety behaviours and rewards [45]. Safey behaviours refer avoidance behaviours used to reduce anxiety and fear (e.g. repeatedly checking locks, leaving on lights). Specific exposure examples provided included gradually increasing time spent alone in bed, reducing parental presence at bedtime with transitional and graded steps.

Several psychologists used play-based and imaginative elements to enhance child engagement.

“[I] *just love the playful element.” – Psychologist4*.

“*Something that I have found very helpful for the kids that I’ve worked with is a clip from Harry Potter about the Boggarts where it’s something scary that comes out from the cupboard and I guess we work with that with the nighttime fear and try and cast a ridiculous spell onto it and make it funny.” – Psychologist3*.

##### Theme 2: parent role and training

Psychologists stressed the importance of parents’ roles in delivering interventions and supporting children with their nighttime fears, emphasising that parental involvement and buy-in often predicted intervention success. One psychologist explained:

*“We found working with parents*,* particularly if we’re talking younger [children]… If we didn’t have the parents on board*,* the success rate wasn’t great.” – Psychologist4*.

The importance of parent training and education, including improving parent-child communication and self-regulation strategies, was also raised. Several psychologists highlighted the use of videos to model parent-child communication and interactions. The use of videos to deliver this were recommended:

*“The reason I talk about videos is the idea that I think it’s difficult for people to know how they’re delivering things*,* and how they’re coming across without seeing how else it might be done… I think you’ve got an opportunity here through the [digital] medium that you’re using.” – Psychologist4*.

*“I think videos is a great idea*,* like actually watching somebody do it” – Psychologist5*.

#### Theme 3: supporting parents

The group identified challenges related to parent’s emotional responses which may maintain the child’s nighttime fears, such as difficulties withholding excessive reassurance and enabling safety behaviours. Emotional regulation strategies for parents were considered essential.

*“Some grounding… something that grounds them into*,* “What is my role here? And how do I want to show up? And how do I regulate myself in order to show up as the parent I want to be?”” – Psychologist3*.

*“For the parents who become distressed or give in*, *maybe normalizing that*,* and where that’s coming from*,* but reiterating the rationale for what we’re doing*,* and that [it is] in the child’s best interest to stick with the program in those moments.” – Psychologist2*.

*“A care plan*,* or maintenance plan… normalizing that there are going to be barriers… potential grounding statements…. and self care or stress management strategies. For when those barriers are going to come up*,* but in one place*,* because we know that there’s going to be those times where things are much more exhausting and difficult. And so if they can pull up a single screen.” – Psychologist1*.

Psychologists highlighted the importance of normalising difficulties and providing concrete supports to help facilitate parental involvement through exposure therapy. Psychologists said:

*“It’s a discrete commitment that is going to be a big impact on their life*,*” – Psychologist2*.

*“It’s a bit like having a newborn again. Who else can you call on? …It’s trying to in some way get the message across - this is as significant as having a kid in hospital.” – Psychologist4*.

One psychologist noted that this is a common barrier to engagement:

*“Parents struggling to manage their own anxiety or their own feelings around*,* withholding reassurance and safety behaviours. And then not really sticking or altering exactly what the decided stepladder step is for the week*,* because they struggle to implement it. So that’s been probably the most common barrier that I’ve found.” – Psychologist2*.

## Discussion

This study aimed to provide a clinical profile of children with nighttime fears and explore the needs and preferences for a digital parent-led intervention for the treatment of nighttime fears in children. This is the first study to examine the broad clinical profile of children with nighttime fears as well as the first to incorporate co-design methodology in the development of a targeted intervention. Our findings indicated the most prevalent primary diagnosis amongst children with nighttime fears was Separation Anxiety Disorder (24%), followed by Specific Phobia - Natural/Environment type (18%). Sleep disturbances were highly prevalent amongst children with nighttime fears, with 71% falling into the pathological range for disorders of initiating and maintaining sleep. Qualitative findings indicated significant challenges for both children and parents in managing distressing emotions at night and the lack of high-quality care options for nighttime fears. Parents noted the importance of the program setting expectations of their role and providing clear and transparent information. Psychologists emphasised the crucial role of the parent in supporting their children, and highlighted the need to equip them with training and ongoing support.

The majority of children met criteria for a psychiatric diagnosis (79%), averaging three disorders each. Anxiety disorders were the most prevalent, with children meeting criteria for an average of two diagnoses, most commonly Separation Anxiety Disorder and Specific Phobia of the Natural/Environmental type. This suggests that nighttime fears are often associated with significant psychiatric symptoms, predominantly anxiety. These findings align with Kushnir et al. (2014), who reported elevated levels of general fears and other behavioural problems in children with nighttime fears [12]. Unlike prior studies that focused narrowly on the content of nighttime fears and its specific relation to DSM-defined anxiety disorders, the current study explored a larger range of psychiatric diagnoses. Muris et al. reported a substantially lower rate of psychiatric diagnoses [4, 15]. For example, in their 2001 study, only 6% of children met criteria for separation anxiety disorder, 4% for overanxious disorder, 2% for animal phobias, and 1% for environmental phobias. Although Muris et al. used a parent-report interview schedule, they required that the child’s report of the fear content to be directly linked to the specific anxiety disorder, which likely contributed to the lower reported rates [4]. Similarly, in their 2000 study employing a child-report interview schedule and exploring general fears (beyond nighttime fears) in younger children (4–12 years), they found that 49% of children met criteria for at least one anxiety disorder at a subclinical or clinical level, with generalized anxiety disorder (15%), separation anxiety disorder (15%) and specific phobia (13%) being the most common [15].

In both studies, these lower prevalence rates likely reflect the more conservative diagnostic approach and community-based recruitment from schools. The current study recruited a self-selected, convenience sample via online advertisements which may have resulted in an overrepresentation of individuals with heightened clinical symptoms. Nonetheless, there is diagnostic overlap between the current and previous studies in children with nighttime fears, particularly in cases of Separation Anxiety Disorder and Specific Phobia, where nighttime fears may reflect an expression of these disorders.

A large proportion of children (95%) fell into the pathological or borderline range for disorders of initiating and maintaining sleep. These findings are consistent with earlier work in younger children (4–6 years old) showing that nighttime fears are linked to sleep problems, including difficulties in sleeping alone, resistance to going to bed and reduced sleep duration [46]. The authors suggest that nighttime fears may function as both a risk and maintaining factor for disrupted sleep. Similarly, other studies report strong association between anxiety and sleep disturbances. For example, up to 90% of children with Generalised Anxiety Disorder experience at least one sleep-related problem [18, 47]. Several mechanisms have been proposed to account for the comorbidity between anxiety and sleep disturbances [48]. One possible explanation draws on established cognitive and neurocognitive models of sleep disturbance in adults [49–55], which suggest that pre-sleep arousal (e.g., nighttime fears) contribute to difficulties initiating and maintaining sleep. This association between cognitive arousal and sleep disturbances have also been replicated in children [56–58]. Given the temporal proximity of nighttime fears to sleep onset, and consistent with these models, it is unsurprising that children with such fears also report problems initiating and maintaining sleep.

Regarding fear acquisition in the current study, 29% of parents reported that their child had been exposed to fear-inducing television content, while an additional 24% acknowledged this as a possibility (responded “maybe”). These findings are comparable to previous research where 36% of parents identified television as a source of fear acquisition [4]. Given the pervasive nature of media exposure in childhood, these figures likely actual exposure. Importantly, not all media exposure leads to fear development and fear acquisition is understood to be a complex interplay of biological, environmental and cognitive-meditational processes [2, 59].

Although the mechanisms of fear acquisition are beyond the scope of this study, parental *beliefs* about the origins of their fears may offer valuable clinical insight. Prior research has shown that parental interpretations, such as verbal threat information, can influence children’s fear responses [60]. These perceptions may shape how parents respond to their child’s fears, potentially perpetuating and maintaining the parent–child transmission of fear. Future research may benefit from exploring these beliefs to inform treatment approaches that guide parents toward more adaptive interpretations and supportive responses.

Qualitative findings highlight the substantial impact of nighttime fears on both the child’s well-being and overall family functioning. Parents described the significant emotional toll of these nighttime fears, recounting feelings of exhaustion, distress, and guilt. Many reported trying numerous strategies to support their children, yet these efforts were frequently ineffective. The persistence of their child’s fears, along with the financial and occupational impacts, and lack of effective interventions contributed to the parents’ frustration and emotional strain. King et al. similarly describe nighttime fears as highly emotional and disruptive, with many parents ultimately “giv[ing] up and allow[ing] the child to sleep all night sharing the parent’s or siblings; beds” [61]. In the current study, some parents reported using co-sleeping to ease their child’s fears, finding it effective. These positive effects are consistent with attachment theory, which highlights the role of co-regulation [62]. However, many parents expressed their concerns with co-sleeping and the challenges and disruptions associated with reactive co-sleeping. These findings highlight the importance of equipping families with strategies that are both effective and feasible within their daily routines.

Both parents and psychologists emphasised the critical role of parents in the success of a program for nighttime fears in children. Parents highlighted the value of setting clear expectations and fostering a commitment to engage fully with the program. This reflects challenges noted in previous research, which identified issues with parental compliance including misunderstanding instructions or modifying the treatment protocol [63, 64]. These findings align with existing literature in digital sleep interventions for adults, further emphasising the importance of clear and supportive communication and providing a rationale to facilitate engagement [17, 65].

Psychologists similarly noted the importance of parental buy-in, parent training and emotional support strategies. Despite the central role of parents in treatment, previous research has highlighted a lack of literature on parental cognitions, resistance and emotions around child’s sleep and bedtime interactions [8]. This gap is also evident in clinical practice, where family involvement in the treatment of childhood anxiety is often limited. Taken together, parents’ feelings of guilt, their strong desire for clear guidance and expectation-setting, and their fears around the risks of exposure suggest potentially low self-efficacy and elevated parental anxiety. These findings underscore the importance of designing a parent-led digital intervention that is not only evidence-based but also safe, inclusive, and accessible. To prevent potential risks expressed by parents such as exacerbating fears, safeguards will be embedded within the program, including clear guidance, optional clinician support, and mechanisms for monitoring progress.

Accessing good quality care for childhood nighttime fears was described as challenging, with parents facing conflicting information, unclear care pathways and unsatisfactory treatment. These findings highlight the need for stronger inter-disciplinary collaboration amongst medical and mental health practitioners, enhanced training, and approaches that address both physical and psychological aspects of sleep and nighttime fears. Clear guidance and referral options are essential, particularly in light of consistent barriers identified in previous research on childhood anxiety and sleep problems such as limited services, long waitlists, and gaps in provider knowledge ( [66–68]– [10]). In addition, given the high comorbidity between nighttime fears and sleep disturbances, screening for sleep disturbances and disorders in children with nighttime fears is crucial, yet is often overlooked in clinical practice [69].

While the current study reflects practices commonly observed in Western contexts, such as promoting child autonomy and discouraging co-sleeping, it is important note that sleep patterns and co-sleeping norms and expectations vary considerably across cultures. Co-sleeping is a common and accepted practice amongst many cultures globally, including Eastern countries such as India and Japan [70, 71]. These cultural differences highlight the need for flexible and culturally sensitive approaches to sleep interventions. In addition, structural factors such as limited digital literacy and internet connectivity may further impact access to the digital intervention, particularly in low-resource or rural communities. Therefore, it is essential for the intervention to incorporate inclusive design features to accommodate diversity in both family practices and technological contexts.

## Limitations

A limitation of this study is that diagnostic classifications were not confirmed through clinician-administered interviews. Instead, they were derived based on a newly developed instrument (ADIS-P-COMP), adapted from the gold-standard *Anxiety Disorders Interview Schedule for Children and Parents (ADIS-C/P*). While this approach offers a streamlined and feasible method for diagnostic assessment, the psychometric properties of the instrument are yet to be formally evaluated. In addition, sleep disturbances were assessed using a questionnaire and not confirmed through clinical interviews or diagnostic or measurement tools. This may not capture the full clinical picture nor differentiate between normative sleep issues and clinically significant sleep disorders.

Another limitation concerns potential self-selection bias. Parents with children experiencing greater distress from nighttime fears may have been more motivated to participate. This self-selecting sample may have more clinically elevated symptoms compared to community samples. In addition, prior research has indicated that a significant proportion of children - up to one-third - have not disclosed their nighttime fears, underscoring the likelihood that families with undetected difficulties may be underrepresented [1]. As a result, the generalisability of the findings may be limited to the broader population.

This study comprised a relatively small sample size (*N* = 34) for the online assessment battery, which may restrict the generalisability of the findings. The limited number may not fully capture the diversity of experiences across the population. While the sample size and potential self-selection bias may limit broader generalisability, the findings are particularly relevant to the intended target group - children aged 7–12 experiencing clinically significant nighttime fears - whose parents are actively seeking support. This alignment strengthens the applicability of the results to the population the program is designed to target.

## Future research

Future research would benefit from exploring the clinical profiles of children with nighttime fears in larger and more diverse samples. Given the high rates of nighttime fears, anxiety and sleep disturbances observed, longitudinal studies to help elucidate the developmental trajectory and causal relationships would advance the field. The novel diagnostic questionnaire (ADIS-5-P-COMP) demonstrates potential given its ease of use and streamlining of the diagnostic process, and further validation studies are warranted. In addition, future research would benefit from exploring the type of assessment feedback that parents and children prefer to receive. While this was not a specific focus of the current study, it is particularly relevant in younger populations where fears may fluctuate over time and present in diverse ways. Future research could incorporate more precise that better capture the temporal sequence of fear acquisition and onset, such as structured parent-child interviews, to reduce ambiguity inherent in retrospective reporting. Striking the right balance between ensuring timely access to appropriate support and avoiding the risk of over-pathologising normal developmental experiences is critical. Finally, further work would benefit from exploring barriers related to help seeking and consideration of various models to support pathways to care to help families access high-quality support.

## Conclusion

The majority of children with nighttime fears present with anxiety disorders, most commonly Separation Anxiety Disorder and Specific Phobia - Natural/Environment type, with comorbidities frequently observed. This co-design process has highlighted the substantial impact of nighttime fears on children and family functioning and validated the critical need for a digital parent-led intervention. Parents reported the lack of help and treatment options available and an interest for a program that clearly defines expectations for their active role in treatment and provides them with transparent guidance. Psychologists echoed the importance of parental involvement in treatment, and the need for parental training and support. These insights will directly inform the development of a digital parent-led treatment program for children with nighttime fears.

## Supplementary Information

Below is the link to the electronic supplementary material.

Supplementary Material 1. Example Interview and Focus Group Questions. A series of example questions asked in the qualitative methods (interview and focus group).

Supplementary Material 2. Evaluating the current study against the Consolidated Criteria for Reporting Qualitative Research checklist (COREQ). This table provides a summary of the current study evaluated against the COREQ checklist.

Supplementary Material 3. Parent-report items assessing nighttime fears.

## Data Availability

The datasets used and/or analysed during the current study are available from the corresponding author on reasonable request.
