# Supplementary material for: Nighttime fears in children: clinical characterisation and the user needs and preferences for a digital parent-led intervention
Source: Child Adolesc Psychiatry Ment Health. 2026 Jan 21;20:22. doi: 10.1186/s13034-025-01011-2 (PMC12905873; doi:10.1186/s13034-025-01011-2)
Supplement: Supplementary file 1 — Supplementary Material 1. Example Interview and Focus Group Questions. A series of example questions asked in the qualitative methods (interview and focus group). [file 13034_2025_1011_MOESM1_ESM.docx]

**Additional File 1**

**Example Interview and Focus Group Questions**

**Interview Phase 1**

- Could you tell us what your child is afraid of at night?
- Now, can you think of a typical example in the last week where your child was afraid at night?
- What strategies have you tried for your child’s nighttime fears? What worked? What didn’t work?
- How does your child’s nighttime fears impact you and the family?
- What have been the biggest barriers to success in helping your child with their night-time fears?
- What would you like to see in an intervention program for children with nighttime fears?
- What is your knowledge around approaches and treatments for night-time fears in children?
- How else could we make the program fun (for child) to use?
- What additional support do you think would be helpful for parents to support their child’s fears?

**Interview Phase 2**

- Facilitator shows parent prototype or mock-up of nighttime fears intervention

Example prompt questions:

- What do you think about this exercise/feature?
- What do you think could be improved?
- What would you change?
- Can you explain the reason or value of…?

**Focus group**

- How would you usually go about treatment for nighttime fears?
- If parents were to facilitate this therapy, what would the most crucial pieces of support, knowledge or training you think they would need?
- What other exposure activities/games have you used in the past?
- After presenting exposure activity ideas - What are your initial thoughts or questions you have in seeing this?
- What support do you think parents would need to facilitate these activities?
