# Supplementary material for: Nighttime fears in children: clinical characterisation and the user needs and preferences for a digital parent-led intervention
Source: Child Adolesc Psychiatry Ment Health. 2026 Jan 21;20:22. doi: 10.1186/s13034-025-01011-2 (PMC12905873; doi:10.1186/s13034-025-01011-2)
Supplement: Supplementary file 2 — Supplementary Material 2. Evaluating the current study against the Consolidated Criteria for Reporting Qualitative Research checklist (COREQ). This table provides a summary of the current study evaluated against the COREQ checklist. [file 13034_2025_1011_MOESM2_ESM.docx]

Additional File 2

Evaluating the current study against the Consolidated Criteria for Reporting Qualitative Research checklist (COREQ)

| **Item** | **Question/topic** | **Comment** (page numbers from Manuscript file) |
| --- | --- | --- |
| **Domain 1: Research team and reflexivity** | | |
| **Personal Characteristics** | | |
| 1 | Interviewer/facilitator  Which author/s conducted the interview or focus group? | MA and conducted all stage 1 interviews.  SC conducted all stage 2 interviews.  MA and SC jointly facilitated the focus group. (Page 6) |
| 2 | Credentials  What were the researcher’s credentials? E.g. PhD, MD | MA is a Postdoctoral Research Fellow and Clinical Psychologist (MClinPsych, PhD).  SC is a User Experience Researcher. |
| 3 | Occupation  What was their occupation at the time of the study? |  |
| 4 | Gender  Was the researcher male or female? | MA is female.  SC is male. |
| 5 | Experience and training  What experience or training did the researcher have? | MA has prior experiencing in qualitative research across multiple previous studies and has undertaken further training in qualitative research through an additional unit at the University of Sydney.  SC has prior experience in qualitative research on various studies at the institute. SC has completed the User Experience design immersive course completion (General Assembly) |
| **Relationship with participants** | | |
| 6 | Relationship established  Was a relationship established prior to study commencement? | For stage 1 interviews and the focus group, after participants expressed interest in participating in the study, MA contacted participants to provide further information about the study and inviting them to complete the screening questionnaire. After participants completed the questionnaire, eligible participants were emailed with the consent form.  MA completed the informed consent process at the beginning of the interview with parents. Rapport was built at these points of contact.  For second stage of interview, MA introduced participants to SC via email for stage 2 interviews, and SC organised scheduling of these interviews. |
| 7 | Participant knowledge of the interviewer  What did the participants know about the researcher? e.g. personal goals, reasons for doing the research | Participants were aware that the data will contribute to the authors development of an intervention for nighttime fears. |
| 8 | Interviewer characteristics  What characteristics were reported about the interviewer/facilitator? e.g. Bias, assumptions, reasons and interests in the research topic | Participants were aware that MA has a research interest in nighttime fears.  Participants were aware of the occupations of the interviewers (MA: Clinical Psychologist and SC: User Experience Researcher) |

| **Domain 2: study design** | | |
| --- | --- | --- |
| **Theoretical framework** | | |
| 9 | Methodological orientation and theory  What methodological orientation was stated to underpin the study? e.g. grounded theory, discourse analysis, ethnography, phenomenology, content analysis | Thematic framework analysis (Page 9) |
| **Participant selection** | | |
| 10 | Sampling  How were participants selected? e.g. purposive, convenience, consecutive, snowball | Convenience Sampling (Page 6) |
| 11 | Method of approach  How were participants approached? e.g. face-to-face, telephone, mail, email | Recruitment included social media advertising and emails to the authors’ network of psychologists. A database of participants with lived experience were also approached via email. (Page 6) |
| 12 | Sample size  How many participants were in the study? | N = 44  Online Assessment Battery: n=34  Interviews: n=5  Focus group: n=5 |
| 13 | Non-participation  How many people refused to participate or dropped out? Reasons? | Nil drop outs |
| **Setting** | | |
| 14 | Setting of data collection  Where was the data collected? e.g. home, clinic, workplace | Zoom interviews and focus group were conducted from a private room, either in an office or home setting |
| 15 | Presence of non-participants  Was anyone else present besides the participants and researchers? | No |
| 16 | Description of sample  What are the important characteristics of the sample? e.g. demographic data, date | See Participant demographics (Page 11, 15 and Additional File 3) |
| **Data collection** | |  |
| 17 | Interview guide  Were questions, prompts, guides provided by the authors? Was it pilot tested? | See Additional File 1 for example interview and focus group questions. All interview and focus group guides were developed and/or reviewed by at least two researchers including senior author (JH). |
| 18 | Repeat interviews  Were repeat interviews carried out? If yes, how many? | Repeat interviews were conducted with five parents, as the interview process was carried out in two stages. |

| 19 | Audio/visual recording  Did the research use audio or visual recording to collect the data | All interviews and focus group were audio and video recorded (page xx) |
| --- | --- | --- |
| 20 | Field notes  Were field notes made during and/or after the interview or focus group? | Field notes were documented for all interviews and focus group (page xxxxxx) |
| 21 | Duration  What was the duration of the interviews or focus group? | The average duration of the interviews and focus groups were 54 and 58 minutes, respectively. (page xxxx) |
| 22 | Data saturation  Was data saturation discussed? | Data saturation was reached following the first three interviews with parents (stage 1) and the first two interviews (stage 2). While data saturation could not be assessed due to the single focus group, the session provided in-depth insights aligned with themes from other data sources (page XXXX). |
| 23 | Transcripts returned  Were transcripts returned to participants for comment and/or correction? | Transcripts were not returned to participants for  Correction. |
| **Domain 3: analysis and findings** | | |
| **Data analysis** | | |
| 24 | Number of data coders  How many data coders coded the data? | All interviews were coded by MA. A second researcher (NP, CL) independently double-coded the full first phase of parent interviews and focus group, and 40% of the second phase of interviews (prototype review). (page XXXX) |
| 25 | Description of the coding tree  Did authors provide a description of the coding tree? | Yes, see Results.(page XXX) |
| 26 | Derivation of themes  Were themes identified in advance or derived from the data? | Themes were derived from the data as per Stage 2 of framework analysis (page xxxx) |
| 27 | Software  What software, if applicable, was used to manage the data? | NVivo 12 (page zz) |
| 28 | Participant checking  Did participants provide feedback on the findings? | Participants were not asked to provide feedback on  the findings |
| **Reporting** | | |
| 29 | Quotations presented  Were participant quotations presented to illustrate the themes / findings? Was each quotation identified? e.g. participant number | Yes, see Results |
| 30 | Data and findings consistent  Was there consistency between the data presented and the findings? | Yes, see Results |
| 31 | Clarity of major themes  Were major themes clearly presented in the findings? | Yes, see Results |
| 32 | Clarity of minor themes  Is there a description of diverse cases or discussion of minor themes? | Yes, see Results |
